# Cancer incidence in the East Azerbaijan province of Iran in 2015–2016: results of a population-based cancer registry

**DOI:** 10.1186/s12889-018-6119-9

**Published:** 2018-11-19

**Authors:** Mohammad Hossein Somi, Roya Dolatkhah, Sepideh Sepahi, Mina Belalzadeh, Jabraeil Sharbafi, Leila Abdollahi, Azin Nahvijou, Saeed Nemati, Reza Malekzadeh, Kazem Zendehdel

**Affiliations:** 1 Liver and Gastrointestinal Diseases Research Center, Tabriz University of Medical Sciences, Tabriz, Iran; 2 Hematology and Oncology Research Center, Tabriz University of Medical Sciences, Tabriz, Iran; 3 Cancer Registry Office, Tabriz University of Medical Sciences, Tabriz, Iran; 4 Cancer Research Center, Cancer Institute of Iran, Tehran University of Medical Sciences, Tehran, Iran; 5 Digestive Oncology Research Center, Digestive Disease Research Institute, Tehran University of Medical Sciences, Tehran, Iran; 6 Cancer Biology Research Center, Cancer Institute of Iran, Tehran University of Medical Sciences, Tehran, Iran

**Keywords:** Cancer, Registry, Population-based, Iran, Incidence

## Abstract

**Background:**

Few countries in the Middle East have a population-based cancer registry, despite a clear need for accurate cancer statistics in this region. We therefore established a registry in the East Azerbaijan province, the sixth largest province in northwestern Iran.

**Methods:**

We actively collected data from 20 counties, 62 cities, and 44 districts for the period between 20^th^ March 2015 and 19^th^ March 2016 (one Iranian solar year). The CanReg5 software was then used to estimate age-standardized incidence rates (ASRs) per 100,000 for all cancers and different cancer types.

**Results:**

Data for 11,536 patients were identified, but we only analyzed data for 6655 cases after removing duplicates and non-residents. The ASR for all cancers, except non-melanoma skin cancer, was 167.1 per 100,000 males and 125.7 per 100,000 females. The most common cancers in men were stomach (ASR 29.7), colorectal (ASR 18.2), bladder (ASR 17.6), prostate (ASR 17.3), and lung (ASR 15.4) cancers; in women, they were breast (ASR 31.1), colorectal (ASR 13.7), stomach (13.3), thyroid (ASR 7.8), and esophageal (ASR 7.1) cancers. Both the death certificate rate (19.5%) and the microscopic verification rate (65%) indicated that the data for the cancer registry were of reasonable quality.

**Conclusion:**

The results of the East Azerbaijan Population-based Cancer Registry show a high incidence of cancer in this province, especially gastrointestinal cancers.

## Background

Cancer is a major cause of mortality and morbidity worldwide. According to the GLOBOCAN project, there were an estimated 14.1 million new cases and 8.2 million deaths from cancer globally in 2012 [1]. In 2013, the cancer incidence increased to 14.9 million new cancer cases [2]. Programs for population-based cancer registration are considered the gold standard method for collecting data on all new cancer cases. Moreover, these programs are essential for estimating the burden of cancer in specific populations, particularly when seeking to provide a framework for clarifying community-based risk factors and monitoring efforts to control cancer [2–4]. A National Pathology-based Cancer Registry Program was started in Iran in 2001 and was subsequently rolled out to all provinces, including the East Azerbaijan province [5]. However, there were large differences in the coverage of cancer registry program in all state and provinces of Iran, which made this program cover about 60-70% of all cancer cases data. [5–9] Etemadi et al. has described the numerous attempts to establish a population-based cancer registry in Iran [9, 10]. One registry was maintained in the Golestan province [11–13], and its most recent results were published in the International Agency for Research on Cancer (IARC) monograph, “Cancer in Five Continents,” in 2013 [14].

The first population-based studies in Iran estimated the incidence rates of all cancer types during 1996–2000 in the Golestan, Mazandaran, and Kerman provinces [15], for the Ardabil Province for 1996–1999 [7], and for the East Azerbaijan province for 2006–2007 [16]. The Iranian national population-based cancer registration is following a five-year plan, covering 14 provinces in its first phase, and is funded by the involved universities. The East Azerbaijan Population-based Cancer Registry (EA-PBCR) was established in 2006-2007, based on the goals and responsibilities directed by the chancellor of Tabriz University of Medical Sciences. In the EA-PBCR, high rates were reported for gastric cancer (ASR 37.6 per 100,000) and esophageal cancer (ASR 24.1 per 100,000) in men, and high rates were reported for breast cancer (ASR 23.5 per 100,000) in women [16]. Stomach cancer was the most common cancer in the north and northwest of Iran [17], as well as in the Ardabil Province for both men and women, and this region has since been considered a high-risk region for stomach cancer [18]. Indeed, according to Marzban et al., death from stomach cancer is up to six-fold higher in this region compared with southern Iran [7, 19].

In this study, we aimed to establish a standard population-based cancer registry in the East Azerbaijan province. The component aims were to create the necessary infrastructures, to initiate networking and collaborations between data sources, and to estimate the incidence rates for different cancers in this province. Research grants were obtained for stable funding, and we ensured proper governance and agreement among stakeholders. In this paper, we report the first results of the EA-PBCR.

## Methods

### Data source

Well-trained health information technology staff (the EA-PBCR team) collected data from 20 counties, 62 cities, and 44 districts about newly diagnosed cancers between March 20, 2015, and March 19, 2016 (one Iranian solar year). Sources included the following: pathology reports from 33 centers (including pathology and cytology reports); the medical records from 20 educational and private hospital, radiotherapy, and hematology centers; and 35 imaging centers. In several pathology laboratories and hospitals, data were obtained electronically from their information technology systems. Data for cancer-related deaths were obtained from death certificate registry data stored at Tabriz University of Medical Sciences. Also, we linked the last three years data of the national pathology-based cancer registry of Iran (INPCR), which led to the removal of additional duplicate records.

Data were imported to CanReg5 software (Persian Version), which is an open-source tool that allowed information input, storage, checking, and processing. The data were then divided into patient, tumor, and source tables. The following information was mandatory for inclusion in the study: first and last name, birth date, fathers name, national identification (NID) number, sex, place of residence, and date of diagnosis. The morphology (i.e., histology, behavior, and grade) and topography (primary site of origin) of the tumor, based on the International Classification of Diseases for Oncology Third Edition (ICD-O-3), were also reported [20]. Contact information was available for about 75% of cases, which will allow direct access to individual data in future studies. We then completed data entry, quality control, consistency checks, and basic analysis, using the Persian Version of CanReg5.

### Population of East Azerbaijan Province

**Fig. 1:**
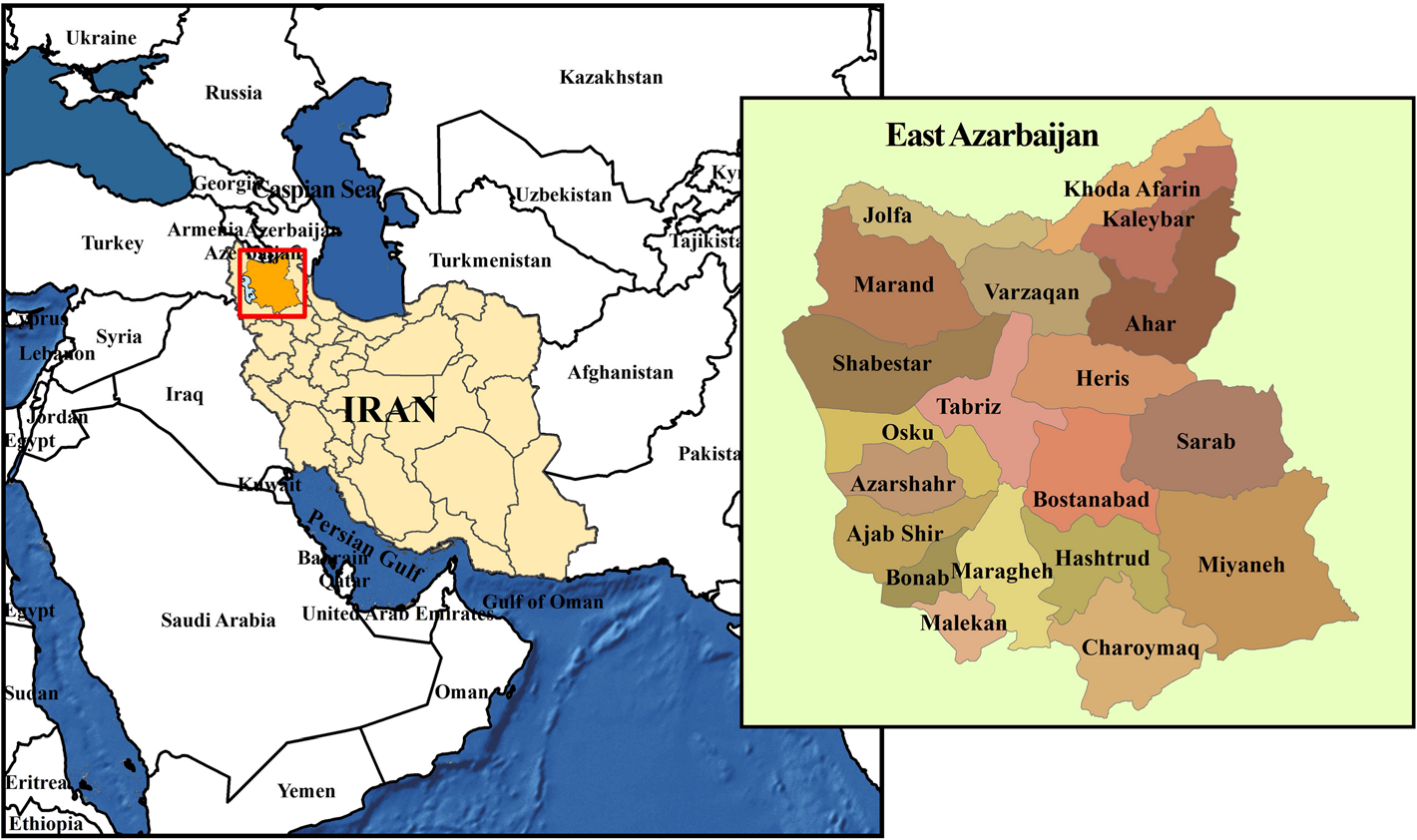
Map of Iran showing East Azerbaijan (cities are shown in the inset)

The East Azerbaijan province is one of 31 provinces of Iran and is the biggest and most populated province of northwest Iran (Fig. 1). It has the sixth largest population overall, and the largest Azeri ethnic population, in Iran. It covers an area of 45,620 km^2^ and had a total population of 3,911,278 according to the 2015 national census in Iran. The EA-PBCR is held in the capital city, Tabriz.

**Fig. 2:**
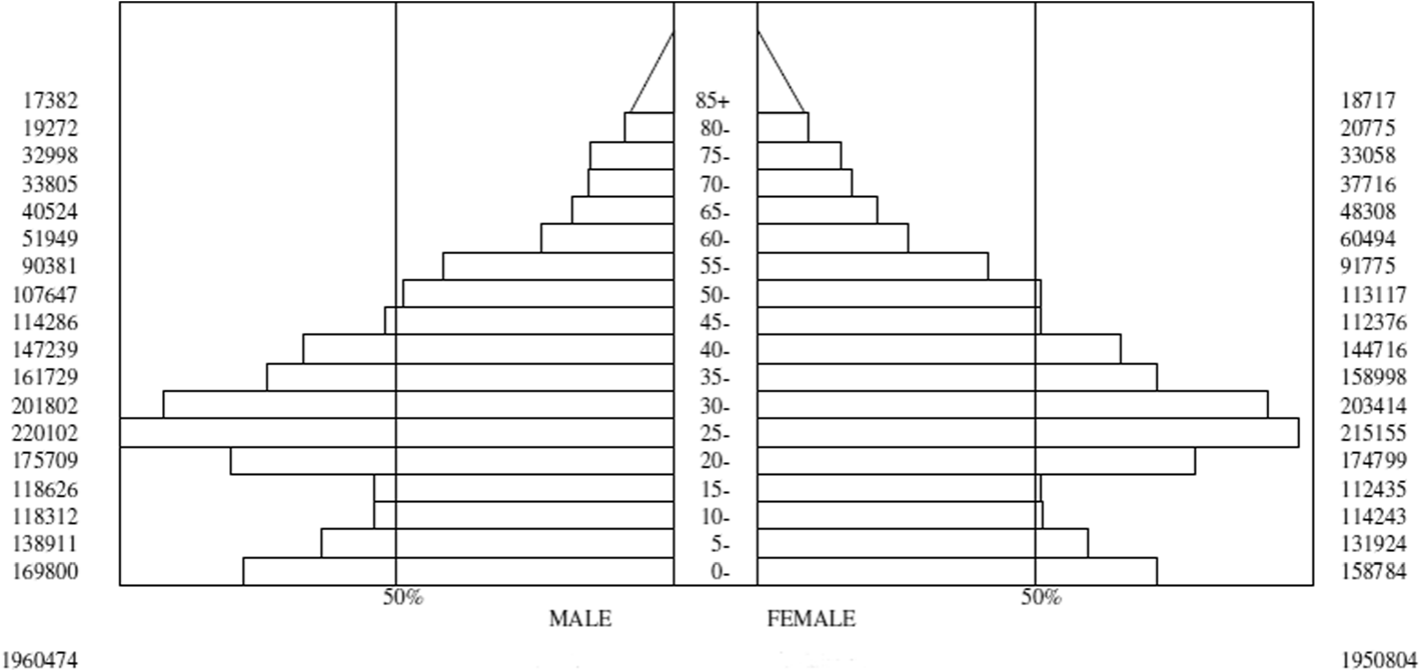
The population distribution pyramid in the East Azerbaijan province, based on the national census report 2015–2016

Figure 2 shows a population pyramid for the East Azerbaijan province by age and sex group, emphasizing that the population was young, with a predominance of people aged 20–24 years. Population density can be seen to reduce gradually with increasing age, such that very few people aged 60–64 years and older were included. Table 1 shows the age-stratification groups in East Azerbaijan during the study period (Table 1, Fig. 2).

**Table 1:** The Exact Stratification Age Groups of East Azerbaijan, in 2015-2016

Age Groups		East Azerbaijan Population in 2015-2016		
	Male	Female		
	Number	Percent	Number	Percent
0-4	169800	8.6	158784	8.13
05-9	138911	7.08	131924	6.76
10-14	118312	6.03	114243	5.85
15-19	118626	6.05	112435	5.76
20-24	175709	8.96	174799	8.96
25-29	220102	11.22	215155	11.02
30-34	201802	10.29	203414	10.42
35-39	161729	8.24	158998	8.15
40-44	147239	7.51	144716	7.41
45-49	114286	5.82	112376	5.76
50-54	107647	5.49	113117	5.79
55-59	90381	4.61	91775	4.70
60-64	51949	2.64	60494	3.10
65-69	40524	2.06	48308	2.47
70-74	33805	1.72	37716	1.93
75-79	32998	1.68	33058	1.69
80-84	19272	0.98	20775	1.06
85<	17382	0.88	18717	0.95
Total	1960474	100	1950804	100

### Quality control

The CanReg5 software was used to perform automatic checks for internal validity. We also performed quality control by manual and computerized validity checks of the cancer registry system based on the IARC criteria [21] in the cancer registry office of East Azerbaijan province. This involved assessing factors influencing comparability, validity, timeliness, and completeness [22–25]. Case duplication across the registry databases was checked in three steps to exclude repeated cases, as follows: 1) patient first name, family name; 2) patient first name, family name, and fathers’ name; and 3) patient NID number. For multiple primary tumors we referred to IARC multiple primary rules for patients multiple primary cancers [26].

Also we discuss with an expert oncologist and pathologist, to determine primary tumors from invasion, metastasis, or recurrence cancer cases according the morphology and behavior of cancers, to avoid any missed and/or duplicated cancer data.

Microscopic verification (MV) is an important quality indicator for any cancer registry. We therefore automatically collected MV data from all available pathology reports (including histological and cytological reports) in laboratory databases. In some cases, these data were collected manually. Cases obtained from the death certificate registry without pathology or clinical data were reported as death certificate only (DCO) cases. We then aimed to decrease the percentage of DCO cases (i.e., the DCO %). First, we performed data linkage with several sources to identify histologic or clinical information. Second, we contacted patients or their relatives to increase the frequency of cases with MV or clinical data. Next, the incidence and mortality rates in the EA-PBCR for the period of study were used to calculate the mortality-to-incidence (M/I) ratio. The mortality data for this analysis were collected from our cancer registry, observed deaths (reports from follow-up records), or the national death certificate registry (reported deaths).

### Statistical analyses

Descriptive data are presented as means ± standard deviation or as numbers and percentages, unless otherwise stated. We estimated the frequencies, crude incidence rates, and age-standardized incidence rates (ASR) per 100,000 populations for all cancers, as well as for the different cancers among men and women. These ASRs per 100,000 were reported for each cancer in 18 strata of 5-year age groups. The standard world population for 2000 was used to estimate the ASR [27].

## Results

### Study sample

**Fig. 3:**
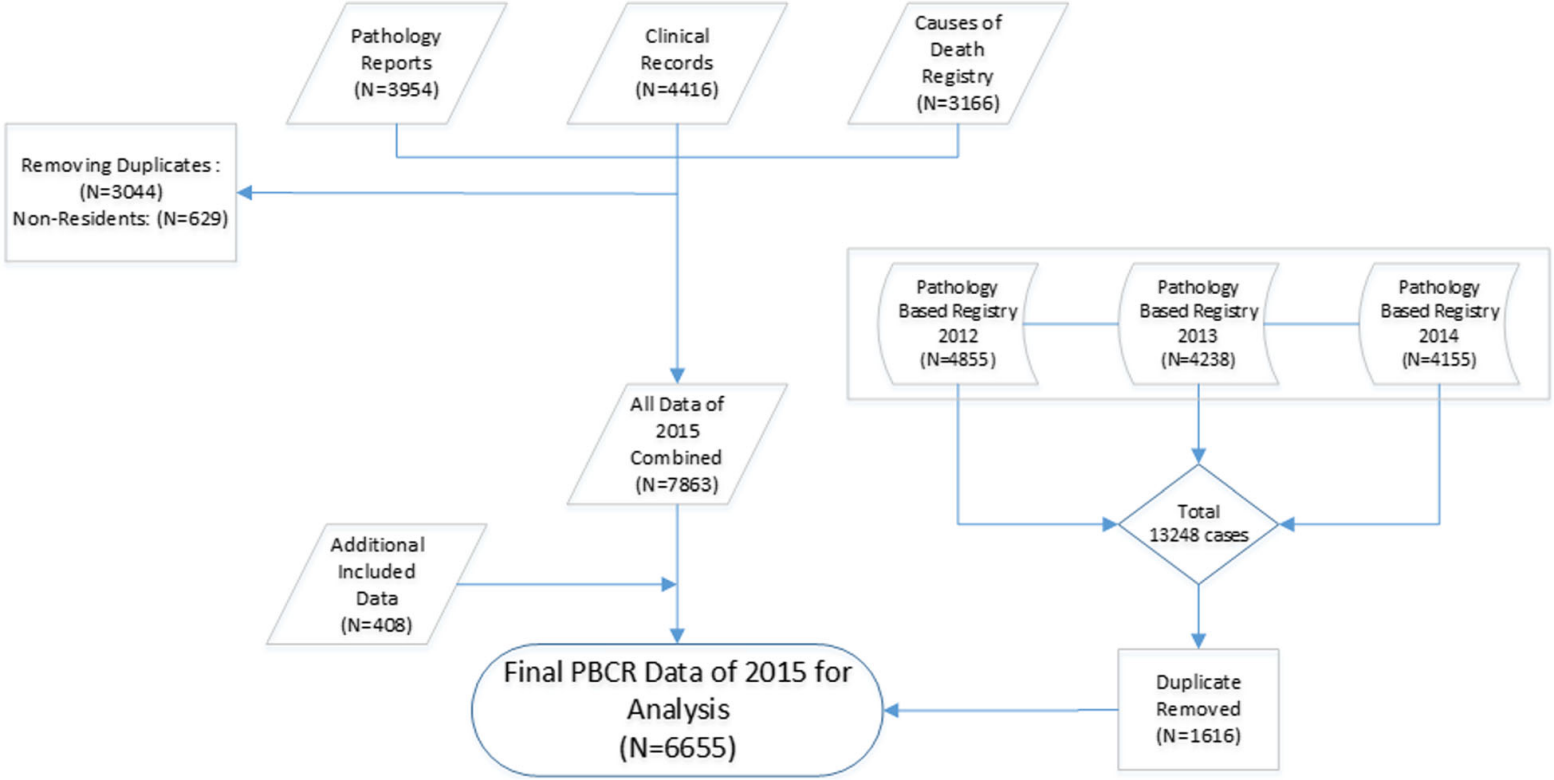
Data linkage and the removal of duplicates and non-residents from the EA-PBCR for 2015–2016

Figure 3 shows the process and role of different data sources in the EA-PBCR. We merged 3954 records from pathology reports with 4416 reports from the medical records of hospital departments and 3166 reports from death certificates (*n* = 11536 cases). After removing 3044 duplicates and 629 records for patients who were referred from neighboring provinces, 7863 records remained. Then, we removed an additional 1616 duplicate records after linkage with INPCR (6247 remained). An additional 408 cases were included during follow-up investigations of patients who were referred to other cities. Finally, the cohort comprised 6655 incident cases that met the inclusion criteria (Fig. 3).

### Data quality

#### Validity and completeness

After rigorous assessment, 65.1% of the cases were found to have MV, including histology (63.2%) and cytology (1.9%) results. Furthermore, 15.2% of reports were collected based on clinical data, including medical records (14%) and imaging reports (1.2%). The remaining data were collected from the cause of death registry (19.5%) and from autopsy records (0.2%), producing a final DCO% of 19.7%.

#### The M/I ratio

The M/I ratio was about 59% (3954/6655), indicating that we received a sufficient number of records from the cause of death registry and that the DCO% was reasonable.

#### Death certificate notification

The total number of cancer deaths in the cause of death registry for East Azerbaijan was 3166. After removing duplicates by linking the database with current and previous cancer registries, we searched for tumor information from alternative sources to improve the validity of our results because the DCO% was high (19.7%). Following this, 1267 cases remained for analysis (1267/6655=19.0%). Data from death certificates and contact with hospitals and patients’ relatives uncovered the clinical or pathology reports for a further 177 patients. These additions allowed us to change the basis of the associated diagnoses ([1267 − 177] / 6655 = 1090 / 6655 = 16.4%). Thus, although the initial data had a higher DCO%, this decreased after linkage with the different databases, after contacting relatives to remove cases diagnosed previously, and after clarifying the clinical and histological information.

### Cancer incidence

Of the 6655 new cancer cases in during the Iranian solar year under study, males accounted for 3728 (56.02 %) and females accounted for 2927 (43.98%), giving a male-to-female ratio of 1.27. The overall mean age was 60.97 ± 17.21 years, but this was higher for men (63.68 ± 16.68 years) and lower for women (57.53 ± 17.27 years).

**Fig. 4:**
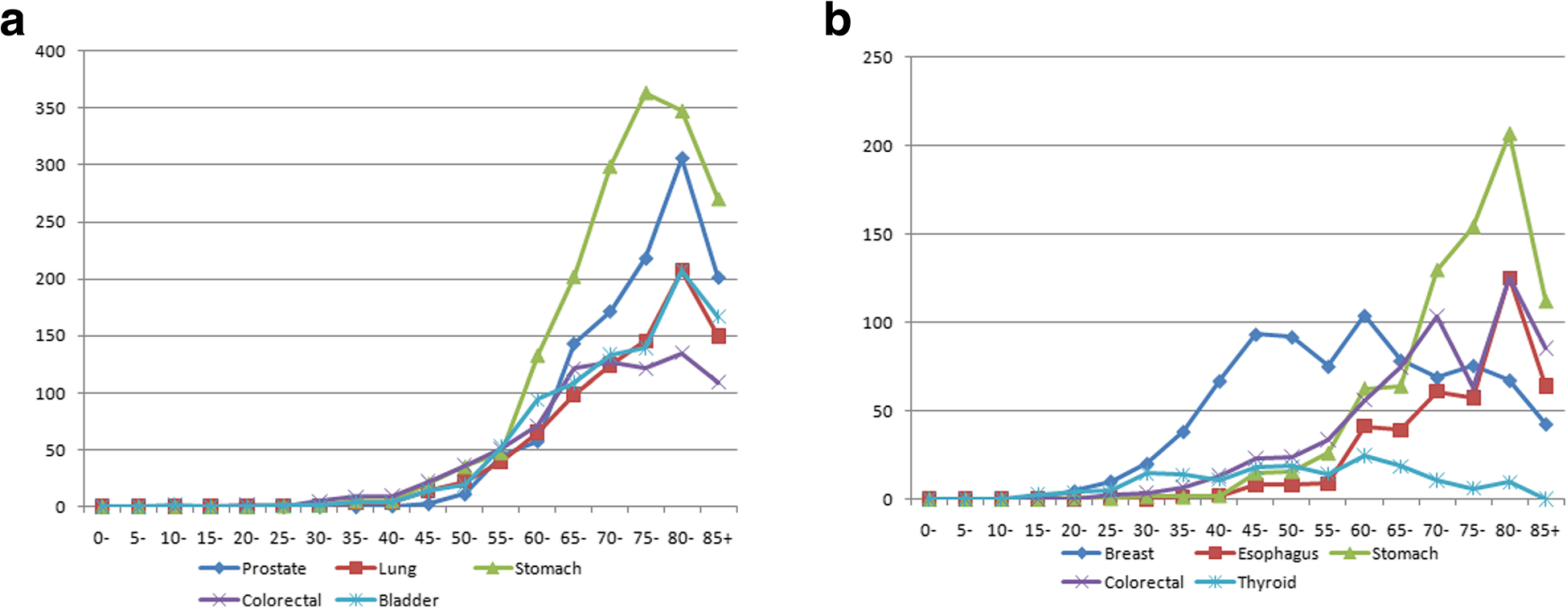
**a** ASRs for the top five major cancers in males. **b** ASRs for the top five major cancers in females

**Fig. 5:**
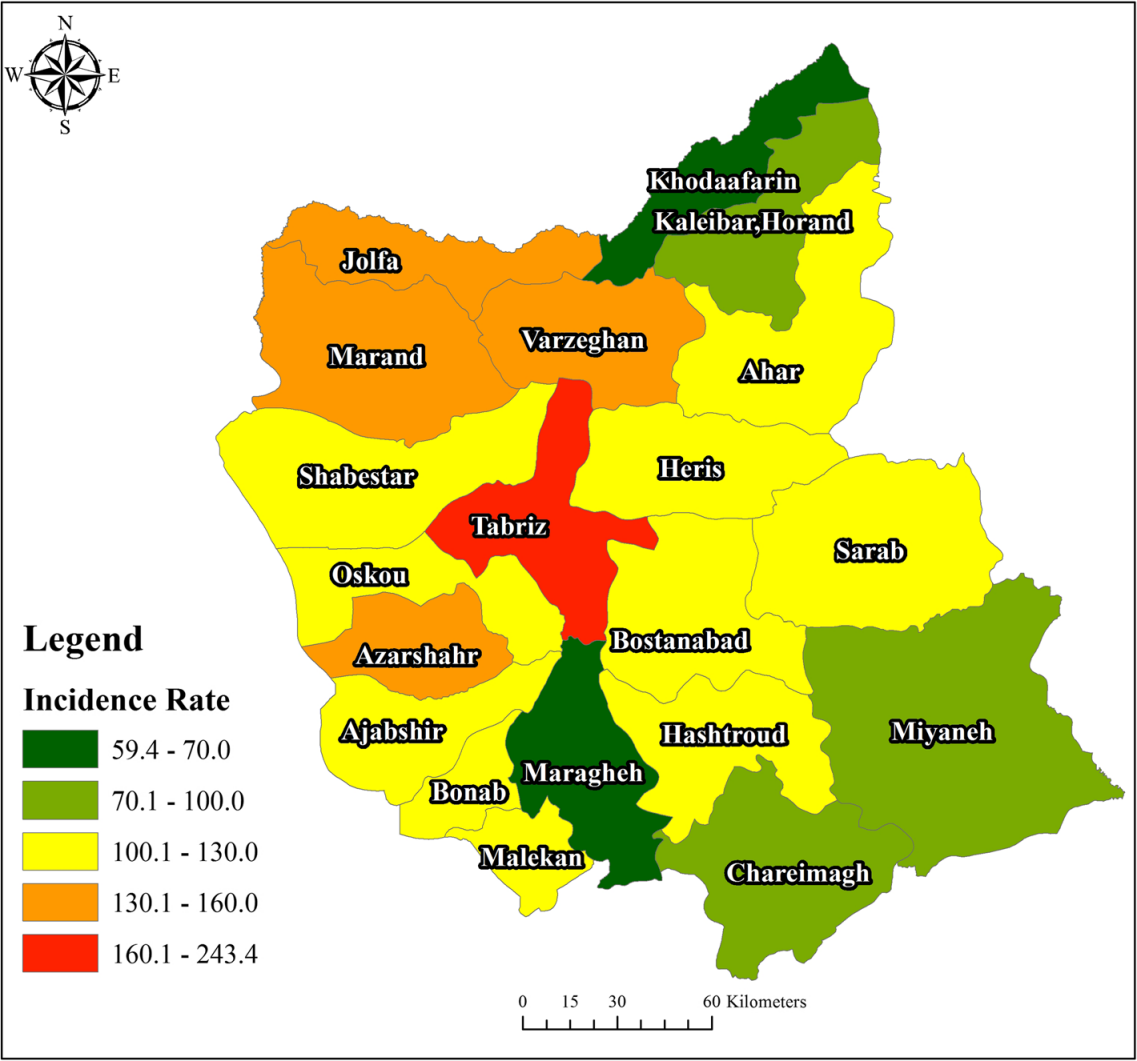
Crude incidence rates of all cancers in East Azerbaijan cities

Except for non-melanoma skin cancer (ICD-O-3 code C44), the crude incidence rates per 100,000 were 175.2 for men and 141.0 for women. The ASRs per 100,000 for all cancers were 167.1 for men and 125.7 for women. The five most common cancers were stomach (ASR 29.7), colorectal (ASR 18.2), bladder (ASR 17.6), prostate (ASR 17.3), and lung (ASR 15.4) for men; and for women, these were breast (ASR 31.1), colorectal (ASR 13.7), stomach (ASR 13.3), thyroid (ASR 7.8), and esophageal (ASR 7.1). Table 2 and Figs. 4, 5 summarize these results, while Table 3 shows the morphologic distributions and frequencies of the most common cancers in the province during 2015–2016 (Tables 2, 3, Figs. 4, 5).

**Table 2:** ASRs for the top ten major cancers in males and females in East Azerbaijan Province in 2015-2016

Male					Female				
Site (ICD-O-3)^a^	No. of Cases	Proportion (%)	CIR^b^	ASR^c^	Site (ICD-O-3)	No. of Cases	Proportion (%)	CIR	ASR
Stomach (C16)	610	17.8	31.1	29.7	Breast (C50)	681	24.8	34.9	31.1
Colorectal (C18-21)	366	10.7	18.7	18.2	Colorectal (C18-21)	299	10.8	15.4	13.7
Bladder (C67)	356	10.4	18.2	17.6	Stomach (C16)	302	11.0	15.5	13.3
Prostate (C61)	369	10.7	18.8	17.3	Thyroid (C73)	175	6.4	9.0	7.8
Lung (C33-34)	320	9.3	16.3	15.4	Esophagus (C15)	157	5.7	8.0	7.1
Leukemia (C91-95)	196	5.7	10	9.6	Leukemia (C91-95)	117	4.2	6	5.5
Esophagus (C15)	172	5.0	8.8	8.0	Lung (C33-34)	117	4.3	6.0	5.0
Lymphoma (C81-85,88,90,96)	126	3.7	6.4	6.4	Ovary (C56)	97	3.5	5.0	4.8
Liver (C22)	115	3.3	5.9	5.9	Bladder (C67)	87	3.2	4.5	3.9
Brain& CNS (C70-72)	111	3.2	5.7	5.3	Liver (C22)	82	3.0	4.2	3.5

^a^International Classification of Diseases for Oncology Third Edition code

^b^Crude Incidence Rate

^c^Age-standardized Incidence Rate

**Table 3:** Morphologic Distribution and Frequency of most common cancers in East Azerbaijan Province in 2015-2016

Cancer Site		Frequent	Percent	Cancer Site		Frequent	Percent	Cancer Site		Frequent	Percent
Cardiac Gastric Cancer	Gender			Esophageal Cancer	Gender			Breast Cancer	Gender		
Neoplasm, Malignant	Male	4	7.0	Neoplasm, Malignant	Male	14	8.1	Neoplasm, Malignant	Male	1	0.14
	Female	2	8.0		Female	9	5.7		Female	43	6.09
Epithelial Tumor, Malignant	Male	1	1.8	Epithelial Tumor, Malignant	Male	4	2.3	Carcinoma	Male	-	-
	Female	-	-		Female	2	1.3		Female	28	3.96
Squamous Cell Carcinoma	Male	1	1.8	Squamous Cell carcinoma	Male	131	75.7	Papillary Carcinoma	Male	1	0.14
	Female	8	32.0		Female	141	88.7		Female	-	-
Adenocarcinoma/NOS	Male	24	42.1	Adenocarcinoma/NOS	Male	9	5.2	Squamous Cell Carcinoma	Male	-	-
	Female	8	32.0		Female	5	3.1		Female	1	0.14
Adenocarcinoma, Intestinal Type	Male	19	33.3	Adenocarcinoma, Intestinal Type	Male	12	6.9	Adenocarcinoma	Male	-	-
	Female	4	16.0		Female	1	.6		Female	1	0.14
Adenocarcinoma, Diffuse Type	Male	4	7.0	Adenocarcinoma, Diffuse Type	Male	-	-	Tubular Adenocarcinoma	Male	-	-
	Female	1	4.0		Female	-	-		Female	1	0.14
Mucinous Adenocarcinoma	Male	1	1.8	Mucinous Adenocarcinoma	Male	1	.6	Mucinous Adenocarcinoma	Male	-	-
	Female	-	-		Female	-	-		Female	1	0.14
Sebaceous Adenocarcinoma	Male	-	-	Sebaceous Adenocarcinoma	Male	-	-	Signet Ring Cell Carcinoma	Male	-	-
	Female	1	4.0		Female	-	-		Female	1	0.14
Signet Ring Cell Carcinoma	Male	3	5.3	Signet Ring Cell Carcinoma	Male	1	.6	Ductal Carcinoma	Male	12	1.6
	Female	1	4.0		Female	-	-		Female	587	83.14
Others	Male	-	-	Others	Male	1	.6	Medullary Carcinoma	Male	-	-
	Female	-	-		Female	1	.6		Female	8	1.13
Total	Male	57	100.0	Total	Male	173	100.0	Lobular Carcinoma	Male	-	-
	Female	25	100.0		Female	159	100.0		Female	17	2.40
Non-Cardiac Gastric Cancer				Colorectal Cancer				Infiltrating Ductal and Lobular Carcinoma	Male	-	-
Neoplasm, Malignant	Male	55	9.9	Neoplasm, Malignant	Male	20	5.4		Female	1	0.14
	Female	31	11.1		Female	33	10.7	Phylloides Tumor Malignant	Male	-	-
Epithelial Tumor, Malignant	Male	7	1.3	Epithelial Tumor, Malignant	Male	1	.3		Female	2	0.28
	Female	6	2.1		Female	-	-	Myeloid Sarcoma	Male	-	-
Squamous Cell Carcinoma	Male	3	.5	Squamous Cell Carcinoma	Male	2	.5		Female	1	0.14
	Female	1	.4		Female	2	.7	Unknown	Male	-	-
Adenocarcinoma/NOS	Male	281	50.4	Adenocarcinoma/NOS	Male	330	89.9		Female	-	-
	Female	147	52.5		Female	254	82.7	Total	Male	14	100.0
Adenocarcinoma, Intestinal Type	Male	155	27.8	Adenocarcinoma, Intestinal Type	Male	-	-		Female	692	100.0
	Female	71	25.4		Female	1	.3	Lung Cancer			
Adenocarcinoma, Diffuse Type	Male	18	3.2	Adenocarcinoma, Diffuse Type	Male	-	-	Neoplasm, Malignant	Male	88	19.86
	Female	6	2.1		Female	1	.3		Female	40	9.02
Mucinous Adenocarcinoma	Male	4	.7	Mucinous Adenocarcinoma	Male	6	1.6	Malignant Tumor, Small Cell Type	Male	2	0.45
	Female	1	.4		Female	3	1.0		Female	-	-
Sebaceous Adenocarcinoma	Male	-	-	Sebaceous Adenocarcinoma	Male	-	-	Malignant Tumor, Spindle Cell Type	Male	-	-
	Female	-	-		Female	-	-		Female	1	0.22
Signet Ring Cell Carcinoma	Male	26	4.7	Signet Ring Cell Carcinoma	Male	4	1.1	Carcinoma	Male	11	2.48
	Female	13	4.6		Female	4	1.3		Female	5	1.12
Others	Male	2	.4	Others	Male	4	1.2	Small Cell Carcinoma, NOS	Male	12	2.70
	Female	3	1.1		Female	6	1.8		Female	3	0.67
Unknown	Male	6	1.1	Unknown	Male	-	-	Non-small Cell Carcinoma	Male	5	1.12
	Female	1	.4		Female	-	-		Female	1	0.22
Total	Male	551	100.0	Total	Male	367	100.0	Squamous Cell Carcinoma	Male	172	38.81
	Female	279	100.0		Female	307	100.0		Female	54	12.18
Thyroid Cancer				Prostate				Adenocarcinoma	Male	27	6.09
Neoplasm, Malignant	Male	4	1.70	Neoplasm, Malignant	Male	19	5.09		Female	9	2.03
	Female	8	3.40		Female	-	-	Carcinoid Tumor, NOS	Male	-	-
Papillary Carcinoma	Male	9	3.82	Carcinoma	Male	78	20.91		Female	4	0.90
	Female	28	11.91		Female	-	-	Bronchiolo-Alveolar Adenocarcinoma, NOS	Male	3	0.67
Squamous Cell Carcinoma	Male	-	-	Carcinoma, Undifferentiated ,NOS	Male	1	0.26		Female	-	-
	Female	1	0.42		Female	-	-	Signet Ring Cell Carcinoma,	Male	1	0.22
Papillary Adenocarcinoma	Male	41	17.44	Squamous Cell Carcinoma	Male	1	0.26		Female	-	-
	Female	101	42.97		Female	-	-	Sarcoma, NOS	Male	-	-
Oxyphilic Adenocarcinoma	Male	1	0.42	Transitional Cell Carcinoma, NOS	Male	3	0.80		Female	1	0.22
	Female	6	2.55		Female	-	-	Spindle cell Sarcoma	Male	1	0.22
Follicular Adenocarcinoma	Male	2	0.85	Papillary Transitional Cell Carcinoma	Male	3	0.80		Female	-	-
	Female	13	5.52		Female	-	-	Total	Male	322	100.0
Insular Carcinoma	Male	-	-	Adenocarcinoma	Male	266	71.31		Female	118	100.0
	Female	1	0.42		Female	-	-	Bladder Cancer	Gender		
Papillary Carcinoma, Follicular Variant	Male	3	1.27	Adenocarcinoma, Intestinal Type	Male	2	0.53	Neoplasm, Malignant	Male	17	3.82
	Female	13	5.53		Female	-	-		Female	2	0.44
Medullary Carcinoma with Amyloid Stroma	Male	1	0.42	Unknown	Male	-	-	Transitional Cell Carcinoma, NOS	Male	217	48.76
	Female	-			Female	-	-		Female	49	11.01
Mixed Medullary Carcinoma	Male	2	.84	Total	Male	373	100.0	Papillary Carcinoma	Male	1	0.22
	Female	-			Female	-	-		Female	1	0.22
Medullary Carcinoma, NOS	Male	-						Squamous Cell Carcinoma	Male	1	0.22
	Female	3	1.27						Female	-	-
Total	Male	62	100.0					Papillary Transitional Cell Carcinoma	Male	117	26.29
	Female	173	100.0						Female	34	7.64
								Adenocarcinoma	Male	2	0.44
									Female	1	0.22
								Carcinoid tumor, NOS	Male	3	0.66
									Female	-	-
								Total	Male	358	100.0
									Female	87	100.0

## Discussion

The EA-PBCR was established to allow accurate estimates of annual statistics in the province by collecting information on incident cases of cancer from various sources. The main data sources in this study were pathology reports, medical records, and death certificate. However, records from additional sources were also used to improve the completeness and validity of the results, including from radiotherapy and chemotherapy departments, imaging facilities, and hematology laboratories. The ASRs for all cancers in 2015, excluding non-melanoma skin cancer, were 167.1 per 100,000 for males and 125.7 per 100,000 for females. Our data show that the five most common cancers in this region, by sex, were stomach, colorectal, bladder, prostate, and lung in men, and were breast, colorectal, stomach, thyroid, and esophageal in women. Importantly, both the DCO% and the MV rate indicate that the EA-PBCR data were of reasonable quality. Our results are also comparable with those of GLOBOCAN 2012 and of the latest report of Golestan Province population-based cancer registry (Table 4), which are the most recent and reliable to have been published for Iran [1, 28].

**Table 4:** Comparing of age standardized incidence rate (ASR) for most common cancers in Iran (GLOBOCAN 2012), Golestan Province Population Based Cancer Registry Results (2004–2013), and East Azerbaijan Population Based Cancer Registry Results (2015–2016)

Cancer Site	Gender	GLOBOCAN 2012 Iran			Golestan Province Population Based Cancer Registry Results (2004-2013)			East Azerbaijan Population Based Cancer Registry Results (2015-2016)		
		Number	% Total	ASR	Number	% Total	ASR	Number	% Total	ASR
Esophagus	Male	2898	6.5	9.0	1221	12.3	21.6	171	5.0	8.9
	Female	2445	6.1	8.0	930	10.5	16.8	157	5.7	8.3
Stomach	Male	6640	14.8	20.6	1576	15.8	27.4	610	17.7	32.9
	Female	3020	7.6	9.7	686	7.8	12.0	301	10.9	15.6
Colorectal	Male	3811	8.5	11.6	911	9.2	14.9	412	12.0	22.7
	Female	3352	8.4	10.5	715	8.1	11.6	332	12.1	17.8
Leukemia	Male	2338	5.2	6.9	817	8.2	12.3	196	5.7	9.6
	Female	1588	4.0	4.7	599	6.7	9.1	117	4.2	5.5
Lymphoma	Male	2611	5.9	7.3	567	5.7	8.0	126	3.7	6.4
	Female	1703	4.2	4.9	333	3.8	4.5	69	2.4	3.6
Lung	Male	3307	7.4	10.3	907	9.1	16.0	322	9.4	17.4
	Female	1581	4.0	5.0	362	4.1	6.2	118	4.3	6.1
Melanoma (Skin)	Male	295	0.7	0.9	661	6.6	11.4	9	0.3	0.4
	Female	239	0.6	0.7	416	4.7	7.1	3	0.1	0.1
Breast	Male	-	-	-	-	-	-	14	0.4	0.7
	Female	9795	24.5	28.1	2121	24.0	29.3	683	24.7	36.0
Ovary	Male	-	-	-	-	-	-	-	-	-
	Female	1637	4.1	4.8	400	4.5	5.7	97	3.5	5.4
Prostate	Male	4111	9.2	12.6	690	6.9	12.0	369	10.7	19.4
	Female	-	-	-				-	-	-
Bladder	Male	4277	9.5	13.2	529	5.3	9.2	356	10.4	19.8
	Female	1066	2.7	3.4				87	3.2	4.6
Brain, CNS	Male	1699	3.8	4.6	475	4.8	6.9	111	3.2	5.9
	Female	1358	3.4	3.9	369	4.2	5.3	69	2.5	3.7
Thyroid	Male	513	1.1	1.4	-	-	-	63	1.8	3.1
	Female	1512	3.8	4.0	-	-	-	173	6.3	8.6
All cancers excl. non-melanoma skin cancer	Male	44838	100.0	134.7	10577	100.0	175.0	3439	100.0	186.0
	Female	39991	100.0	120.1	9230	100.0	142.4	2761	100.0	146.1

The EA-PBCR has followed international standards for data collection and reporting since its inception. For example, we linked available data from different sources to improve the validity and completeness of our results. Indeed, the MV rate, the DCO%, and the M/I ratio were comparable to those reported for middle-income countries [29–32]. Another advantage was that we created a unique NID number as a mandatory item for use by hospitals, cause of death registries, and most laboratories. This number allows for deterministic data linkage and for improvements in data validity and completeness. However, the EA-PBCR is still in its infancy, and we faced some important limitations. The DCO%, for example, was still high and was difficult to decrease in the first year. Also NID was not available for all databases, and we will try to improve this in our next reports.

We believe that the quality indicators will improve rapidly as we move forward. To improve the quality of the data produced from these sources, we have called for greater collaboration with the cause of death registry and with hospital managers.

According to this study, gastric cancer—which is the fourth most common cancer worldwide—was the most common cancer among males (ASR 29.7 per 100,000) and the third most common cancer among females (ASR 13.3 per 100,000). A few studies of the incidence of gastrointestinal cancer have been performed in the East Azerbaijan province [5, 16, 33–35]. In the most recent survey of gastrointestinal cancer in this province between 2007 and 2011, the ASR for gastric cancer was raised in both men (26 per 100,000) and women (11.6 per 100,000), and it was the second-leading cause of death (10.4% of all deaths) [34]. Other research showed that stomach cancer has been the most common cancer in north and northwestern Iran over the past 30 years [17, 18, 36]. The highest ASRs for stomach cancer in men (51.8; 95% CI 47.8–55.8) and women (24.9; 95% CI 21.5–27.2) per 100,000 were reported for the Ardebil province in 2010 [7, 18, 37]. Gastric cancer is also known to be the most common cause of cancer-related death in the country, although there is a reported six-fold geographic variation in mortality rates between northwestern and southern Iran [19]. The elevated risk of stomach cancer incidence and mortality in the northwestern region has been linked to the higher prevalence of *Helicobacter pylori* infection [38, 39], tobacco and opium use, and dietary factors [40–45]. A well-designed case–control study in northern Iran showed a positive association between red meat consumption and the risk of gastric cancer [46].

The incidence rates of bladder cancer (ASR 17.6 per 100,000) and lung cancer (ASR 15.4 per 100,000), which are associated with tobacco smoking and opium use [47, 48], were also high in the male population. Studies in the 1980s in Southern Iran [49] and recent studies from Golestan in northwestern Iran [50] have shown that opium is a more important risk factor for bladder cancer than tobacco. Opium use is also common in East Azerbaijan (unpublished data from a Persian cohort) and may play an important role in the etiology of bladder cancer in this province.

The ASR of lung cancer was higher, based on clinical diagnosis or DCO, in the EA-PBCR than the rate reported for Iran in GLOBOCAN 2012 (i.e., 15.4 versus 10.3 per 100,000) [1]. In the Golestan population-based cancer registry, the incidence of lung cancer was also higher than the Iranian average for males (ASR = 17.5 per 100,000) [14]. Therefore, it appears that both the pathology-based registry [6, 9], and GLOBOCAN 2012 may have underestimated the true incidence of lung cancer in Iran. The ASR of lung cancer was also low among females in both Tabriz (ASR 5 per 100,000) and Golestan (ASR 5.6 per 100,000) [14]. A recent analysis of the “National Surveys of Risk Factor of Non-Communicable Diseases (STEPS)” [sic] showed that the prevalence of cigarette smoking was high in northwestern Iran, and that 23.7% of men in the East Azerbaijan province reported being smokers [51]. By comparison, the prevalence of cigarette smoking is reported to be much lower (12.4%) among men in the Bushehr province of southern Iran, where water pipe smoking is preferred; here, the prevalence of water pipe use was reportedly 10% for men and 14.8% for women [51]. Therefore, the high incidence rates for lung and bladder cancers in the East Azerbaijan province may be linked to cigarette smoking, highlighting the importance of tobacco control measures in this region. Opium use is also an emerging risk factor for both respiratory tract and lung cancers [52], with recent cohort studies from Golestan revealing the important role of opium in such cancers [53].

The incidence rates of colorectal, prostate, and breast cancer are increasing in low- and middle-income countries [54, 55], including Iran [33, 56–58]. This study showed that the incidence rates for colorectal cancer were high for both men (20.2 per 100,000) and women (16.2 per 100,000). Likewise, high incidence rates were identified for breast cancer in women (ASR 31 per 100,000) and for prostate cancer in men (ASR 17.3 per 100,000) in the East Azerbaijan province. The World Health Organization Office in the Eastern Mediterranean Region recently published a series of recommendations for early cancer detection [59], and although they did not recommend the need for organized colorectal or breast cancer screening, they did emphasize the need for early diagnosis through improvements in public awareness and management of symptomatic patients. This recommendation might be valid for now, but we need to be prepared for a more comprehensive colonoscopy and mammography screening program in the future, when the incidence rates for these cancers can be expected to increase, making screening programs the most cost-effective options. Worldwide, prostate cancer screening is not recommended because of the high false-positive rate when using the prostate-specific antigen [59].

Based on the national cancer registry results in Iran, thyroid and laryngeal cancers are among the most common cancers of the head and neck, with oral and thyroid cancers being predominant in females [60]. Our results also showed a higher incidence of thyroid cancer in females (ASR = 7.78); indeed, it was the fourth most common cancer after breast, colorectal, and stomach cancers, with an ASR that was increased compared with a previous report from East Azerbaijan [16]. Significant associations between increased thyroid cancer incidence and lifestyle risk factors, radiation exposure, smoking, and obesity have been reported in a few studies [61–63]. The difference in the pattern of thyroid cancer incidence in our female cohort demands more comprehensive studies, which we have already started. The results of these will be presented soon.

Esophageal cancer may be one of the least common cancers in most countries, but it is the fourth most common cancer in Iran, being the second-leading cause of cancer and cancer-related mortality. The highest incidence rates have been reported for northern provinces [13]. According to the most recent results of studies in this region, there was a significant decrease in the incidence of esophageal cancer [34, 64]. However, we still observed a high incidence of esophageal cancer, especially in females, which was the fifth most common cancer among women. Compared with previous reports from the cancer registry, the ASR for esophageal cancer has shown a decrease in East Azerbaijan over the last decade [16, 34].

Finally, we found that the incidence of gynecological cancers, including ovarian (ASR 4.8 per 100,000), cervical (ASR 1.58 per 100,000), and endometrial (ASR 2.8 per 100,000) cancers, were low in East Azerbaijan. The extremely low incidence of cervical cancer was comparable to that previously reported for the Ardabil Province [7]. The prevalence of human papillomavirus (HPV) infection in women has been reported at 6.1% in the East Azerbaijan province [65], compared with 7% in Iran in general [66]. This comparatively low prevalence may be attributable to the local Muslim culture, in which more people engage in safer sexual behaviors and fewer people have multiple sexual partners [16, 66–68]. Given the low incidence of cervical cancer, neither a cervical screening program nor a HPV vaccination program would be cost-effective in this area. However, sexual behavior is changing in younger generations, and this may lead to an increase in the rate of HPV infection and the risk of cervical cancer in the future. Therefore, regular monitoring of the incidence of HPV infection and cervical cancer is warranted [69].

## Conclusions

In this study, we presented the most current and reliable data for cancer incidence in northwestern Iran. The quality of the EA-PBCR is promising, and we believe that maintaining and developing this registry will establish a high-quality population-based cancer registry in the region. The results from the EA-PBCR could also be used to estimate cancer-specific incidence and mortality rates in Iran and neighboring countries, making it a potentially invaluable resource for the planning and monitoring of cancer control programs and for the delivery of reliable epidemiological research. Moving forward, our main aims will be to perform survival analyses of the most common cancers in East Azerbaijan, to ensure that the registry continues to exist, and to further develop the follow-up system.

## Abbreviations

ASR: Age-standardized incidence rate
CI: Confidence interval
CLIN: Clinical
DCO: Death certificate only
EA-PBCR: East Azerbaijan Population-based Cancer Registry
HPV: Human papillomavirus
IARC: International Agency for Research on Cancer
ICD-O-3: International Classification of Diseases for Oncology, Third Edition
MV: Microscopic verification
NID: National identification number
STEPS: National Surveys of Risk Factor of Non-Communicable Diseases
